# Neoadjuvant serplulimab and SOX chemotherapy for locally advanced gastric cancer: pathological responses and systemic immune signatures from a phase II trial

**DOI:** 10.3389/fimmu.2025.1702737

**Published:** 2026-01-12

**Authors:** Hongjie Zhan, Liang Liu, Mingzhi Cai, Weilin Sun

**Affiliations:** 1 Department of Gastric Surgery, Tianjin Medical University Cancer Institute & Hospital, National Clinical Research Center for Cancer, Tianjin Key Laboratory of Digestive Cancer, Tianjin’s Clinical Research Center for Cancer, Tianjin, China; 2 Department of Biotherapy, Tianjin Medical University Cancer Institute & Hospital, National Clinical Research Center for Cancer, Tianjin’s Clinical Research Center for Cancer, Tianjin, China

**Keywords:** gastric cancer, neoadjuvant immunotherapy, serplulimab, SOX chemotherapy, pathological response, systemic immunity, cytokines, T-cell subsets

## Abstract

**Background:**

Neoadjuvant immunotherapy combined with chemotherapy is an emerging strategy for improving outcomes in patients with locally advanced gastric cancer (LAGC). However, clinical evidence regarding the efficacy of serplulimab plus SOX chemotherapy and its immunologic correlates remains limited.

**Methods:**

In this prospective, single-center, phase II trial, patients with cT3-4N+M0 resectable gastric or gastroesophageal junction adenocarcinoma received three cycles of serplulimab combined with SOX chemotherapy, followed by D2 gastrectomy and adjuvant therapy. The primary endpoints were pathological complete response (pCR) and major pathological response (MPR). Circulating immune markers, including cytokines (e.g., IL-1β, TNF-α) and T-cell subsets (e.g., CD4^+^/Treg ratio), were profiled longitudinally to evaluate immune remodeling during neoadjuvant therapy. The exploratory role of perioperative parenteral nutrition (PN) was also assessed.

**Results:**

Among the 33 enrolled patients, all underwent surgery and achieved R0 resection. The pCR and MPR rates were 21.21% and 36.36%, respectively. The 12-month event-free survival (EFS) rate was 82.20% (68.99-97.94). Patients achieving MPR exhibited elevated preoperative IL-1β levels, a lower CD4^+^/Treg ratio, and a higher Treg/CD8+ ratio, suggesting that systemic immune activation may predict better pathological response. While PN transiently preserved lymphocytes and reduced inflammation preoperatively, it showed no sustained postoperative immune effect.

**Conclusion:**

Neoadjuvant serplulimab plus SOX chemotherapy demonstrates promising efficacy in LAGC, with immunologic remodeling potentially serving as a predictor of treatment response. The identification of non-invasive, blood-based immune biomarkers may help guide future patient selection and therapeutic optimization. The ongoing phase III trial (NCT04139135) will validate the efficacy of this perioperative immunochemotherapy strategy in LAGC.

## 1 Introduction

Gastric cancer (GC) remains a major global health concern, with approximately 968,784 new cases and 660,175 deaths reported in 2022 (1, 2). China bears the highest burden, accounting for nearly 40% of global new cases, with an estimated 358,700 new diagnoses in 2022 (3). Notably, Due to the nonspecific symptoms, such as abdominal pain, anorexia, and weight loss, over 80% of newly diagnosed GC patients in China present with locally advanced disease (LAGC), typically defined as clinical stage T3-4 (cT3/4) and/or node-positive (N+) tumors (4, 5). For these patients, perioperative systemic therapy has become the standard of care. Regimens such as SOX (S-1 and oxaliplatin) have been shown to improve R0 resection rates, pathological response, and long-term outcomes (6, 7). Nevertheless, despite comprehensive perioperative management, the 5-year overall survival for LAGC remains below 50%, underscoring the need for more effective multimodal strategies (8).

Immune checkpoint inhibitors (ICIs), particularly those targeting programmed cell death protein 1 (PD-1), have demonstrated significant efficacy in advanced and metastatic GC, as evidenced by the landmark phase 3 CheckMate 649, KEYNOTE-811, and ORIENT-16 trials (9–11). These promising results have prompted the investigation of ICIs in the neoadjuvant setting for resectable GC, with ongoing phase III trials such as KEYNOTE-585 and MATTERHORN are evaluating neoadjuvant chemoimmunotherapy in this context (12, 13). However, short-term efficacy data suggest variability in pathological responses, indicating that treatment outcomes may depend not only on tumor-intrinsic factors but also on host-related immune competence. One underappreciated determinant of immune responsiveness is the patient’s nutritional and systemic immune status. Malnutrition is highly prevalent among GC patients, often resulting from gastrointestinal symptoms and psychological distress following diagnosis (14). During the perioperative period, nutritional decline can be further exacerbated by treatment-related toxicity and reduced oral intake. Malnutrition has been associated with impaired T-cell function, systemic inflammation, and diminished treatment tolerance, all of which may compromise the effectiveness of immunotherapy (15, 16). Although perioperative nutritional support has shown promise in promoting recovery after surgery, its impact on circulating immune parameters and immunotherapeutic efficacy has not been adequately explored.

In this prospective phase II trial, we explored the efficacy and safety of perioperative serplulimab (anti-PD-1 monoclonal antibody) in combination with SOX chemotherapy in patients with resectable LAGC. In addition to assessing pathological response and immune dynamics across the perioperative course, we further explored whether standardized preoperative parenteral nutritional support could modulate systemic immune status and potentially enhance treatment efficacy. This study provides novel insights into the interplay between nutritional status, systemic immunity, and therapeutic response in the context of perioperative immunotherapy for gastric cancer.

## 2 Methods

### 2.1 Study design and patients

This investigator-initiated, prospective, single-center, exploratory phase II clinical trial conducted at Tianjin Medical University Cancer Institute and Hospital, designed to evaluate the efficacy, safety, and immunologic dynamics of perioperative serplulimab combined with SOX chemotherapy in patients with resectable locally advanced gastric or gastroesophageal junction (GEJ) adenocarcinoma. The study was exploratory and intended to generate hypothesis-generating clinical and translational insights. The trial was conducted in accordance with the principles of the Declaration of Helsinki and the Good Clinical Practice (GCP) guidelines, and the results were reported in adherence to the principles outlined in the CONSORT statement for non-randomized trials. The study protocol was approved by the Institutional Review Board (IRB) of Tianjin Medical University Cancer Institute and Hospital (Approval No. E20230556) and registered at ClinicalTrials.gov (Identifier: NCT06496789).

Eligible patients were aged 18–75 years, with histologically confirmed, previously untreated, and resectable gastric or GEJ adenocarcinoma staged as cT3/4 and/or N+, without evidence of distant metastasis. Staging was based on contrast-enhanced computed tomography (CT) or magnetic resonance imaging (MRI), endoscopic ultrasound, and/or diagnostic laparoscopy. All patients had an Eastern Cooperative Oncology Group (ECOG) performance status of 0–1 and adequate organ function, and were deemed suitable for curative-intent D2 gastrectomy by a multidisciplinary team. Key exclusion criteria included prior systemic therapy or radiotherapy for gastric cancer, history of gastrointestinal perforation or fistula within 6 months prior to enrollment, gastrointestinal bleeding within 2 months prior to enrollment, or any condition deemed by the investigator to confer a high risk of gastrointestinal hemorrhage, and thromboembolic events within 6 months. Additional exclusions were uncontrolled hypertension, and a history of other malignancies within the past two years. All patients provided written informed consent prior to any study-related procedures and were informed of their right to withdraw from the study at any time and for any reason without prejudice.

### 2.2 Treatment protocol

All enrolled patients received three cycles of neoadjuvant therapy comprising serplulimab in combination with SOX chemotherapy, followed by curative-intent surgery. Postoperatively, patients received an additional three cycles of adjuvant serplulimab plus SOX chemotherapy, followed by maintenance monotherapy with serplulimab for up to one year after surgery. Serplulimab was administered intravenously at a fixed dose of 300 mg on day 1 of each 3-week cycle (Q3W) during the neoadjuvant, adjuvant, and maintenance phases, and continued until 1 year postoperatively or until disease progression, unacceptable toxicity, or withdrawal of consent. Chemotherapy followed a Q3W SOX regimen, with oxaliplatin administered intravenously at 130 mg/m² on day 1 and S-1 given orally twice daily (BID) from day 1 to day 14. The dose of S-1 was determined based on body surface area (BSA): 40 mg for BSA ≤1.25 m², 50 mg for BSA between 1.25 and 1.50 m², and 60 mg for BSA >1.50 m². At the discretion of the multidisciplinary team, some patients received preoperative parenteral nutritional (PN) support consisting of fat emulsion, amino acids, and glucose solution. Surgery was scheduled 4–6 weeks after completion of neoadjuvant therapy. All surgical procedures were performed with curative intent and included D2 gastrectomy, in accordance with the Japanese Gastric Cancer Treatment Guidelines.

Follow-up assessments included radiological and laboratory evaluations. Tumor imaging was performed at baseline, prior to surgery, and after completion of adjuvant therapy, and then every 3 months for the first 2 years postoperatively. Laboratory tests were conducted on the same schedule. Tumor response was assessed according to RECIST version 1.1. Follow-up continued until disease progression, death, or withdrawal of consent.

### 2.3 Study endpoints

The primary endpoint of this study was the pathological complete response (pCR) rate, defined as the proportion of patients achieving tumor regression grade (TRG) 1, corresponding to the absence of any residual viable tumor cells in the resected specimen (17, 18). Pathological response was assessed postoperatively by experienced gastrointestinal pathologists according to the TRG classification system. Secondary endpoints included the major pathological response (MPR) rate, R0 resection rate, event-free survival (EFS), objective response rate (ORR), and overall survival (OS). MPR was defined as the presence of residual tumor cells comprising less than 10% of the tumor bed. R0 resection was defined as the complete removal of the primary tumor and regional lymph nodes with no macroscopic or microscopic residual disease at the resection margins. EFS was calculated from the date of treatment initiation until the occurrence of one of the following events, whichever occurred first: disease progression according to RECIST version 1.1, as assessed by investigators (19); disease recurrence confirmed by biopsy; any progression requiring non-protocol therapy during the neoadjuvant or adjuvant treatment period; or death from any cause. Disease progression included both RECIST-defined progression and non-RECIST progression (e.g., as determined by investigator assessment or biopsy results). Progression that precluded surgery or necessitated non-protocol treatment during the neoadjuvant phase was also considered an event for EFS. ORR was defined as the proportion of patients who achieved a complete response (CR) or partial response (PR) to neoadjuvant therapy prior to surgery, as evaluated by investigators using the RECIST version 1.1. OS was defined as the time from treatment initiation to death from any cause.

An exploratory endpoint was to evaluate the impact of standardized preoperative PN support on perioperative immune dynamics and short-term therapeutic efficacy. Specifically, we aimed to investigate whether PN support influenced circulating immune cell subsets, inflammatory cytokine profiles, and pathological response outcomes (pCR and MPR rates). Safety endpoints included the incidence and severity of treatment-related adverse events (TRAEs). AEs were monitored throughout the study and graded according to the National Cancer Institute Common Terminology Criteria for Adverse Events (CTCAE), version 5.0.

### 2.4 Statistical analysis

This exploratory, single-arm, phase II study was powered based on historical data, assuming a 15% improvement in pCR rate with the investigational regimen, a two-sided α of 0.05, 80% power, and an anticipated dropout rate of 10%. The planned enrollment was at least 39 patients. All efficacy and safety analyses were performed in the intention-to-treat (ITT) population, defined as all patients who received at least one dose of study treatment, regardless of whether they proceeded to surgery.

Descriptive statistics were used to summarize baseline demographic and clinical characteristics. Categorical variables were expressed as frequencies and percentages and compared using the chi-squared test or Fisher’s exact test, as appropriate. The distribution of continuous variables was assessed using the Shapiro-Wilk test. Normally distributed variables were reported as means with standard deviations (S.D.) and compared using independent-samples t-tests, while non-normally distributed variables were reported as medians with ranges and compared using the Mann-Whitney U test. The following composite indices were calculated: neutrophil-to-lymphocyte ratio (NLR, absolute neutrophil count/absolute lymphocyte count), platelet-to-lymphocyte ratio (PLR, platelet count/absolute lymphocyte count), systemic immune-inflammation index (SII, platelet count × neutrophil count/lymphocyte count), and prognostic nutritional index (PNI, serum albumin level [g/L] + 5 × absolute lymphocyte count [10^9^/L]). The ORR, pCR rate, and MPR rate were reported along with exact 95% confidence intervals (CIs). EFS and OS were estimated using the Kaplan-Meier method, and medians with 95% CIs were provided. To explore the association between preoperative PN support and short-term efficacy, multivariable logistic regression analyses were constructed using pCR or MPR as the dependent variable, with adjustment for potential confounding variables such as baseline demographics, clinical stage, ECOG performance status, and relevant laboratory parameters.

In the exploratory analyses, we investigated the perioperative dynamics of systemic immune markers and their potential association with treatment response. First, to assess changes in immune-related biomarkers across different timepoints (baseline, post-neoadjuvant/preoperative, and postoperative), intergroup comparisons were performed using appropriate statistical tests based on data distribution and variance characteristics. Specifically, for continuous variables that were normally distributed with equal variances, independent-samples t tests were used; for normally distributed variables with unequal variances, Welch’s t tests were applied; and for non-normally distributed variables, Wilcoxon rank-sum tests were employed. Stratification was conducted by preoperative PN support status and by MPR status to explore whether nutritional intervention or tumor regression response influenced systemic immune changes over time. Second, we examined the correlation between perioperative hematologic indices and pathological tumor regression, as determined by TRG classification, using Spearman’s rank correlation coefficients. These exploratory analyses aimed to elucidate whether systemic immune status and its perioperative modulation by nutritional intervention could serve as predictive markers of therapeutic efficacy.

All statistical tests were two-sided, and a P value <0.05 was considered statistically significant. All analyses were conducted using R software (version 4.3.2) and GraphPad Prism (version 10.1.2).

## 3 Results

**Figure 1:**
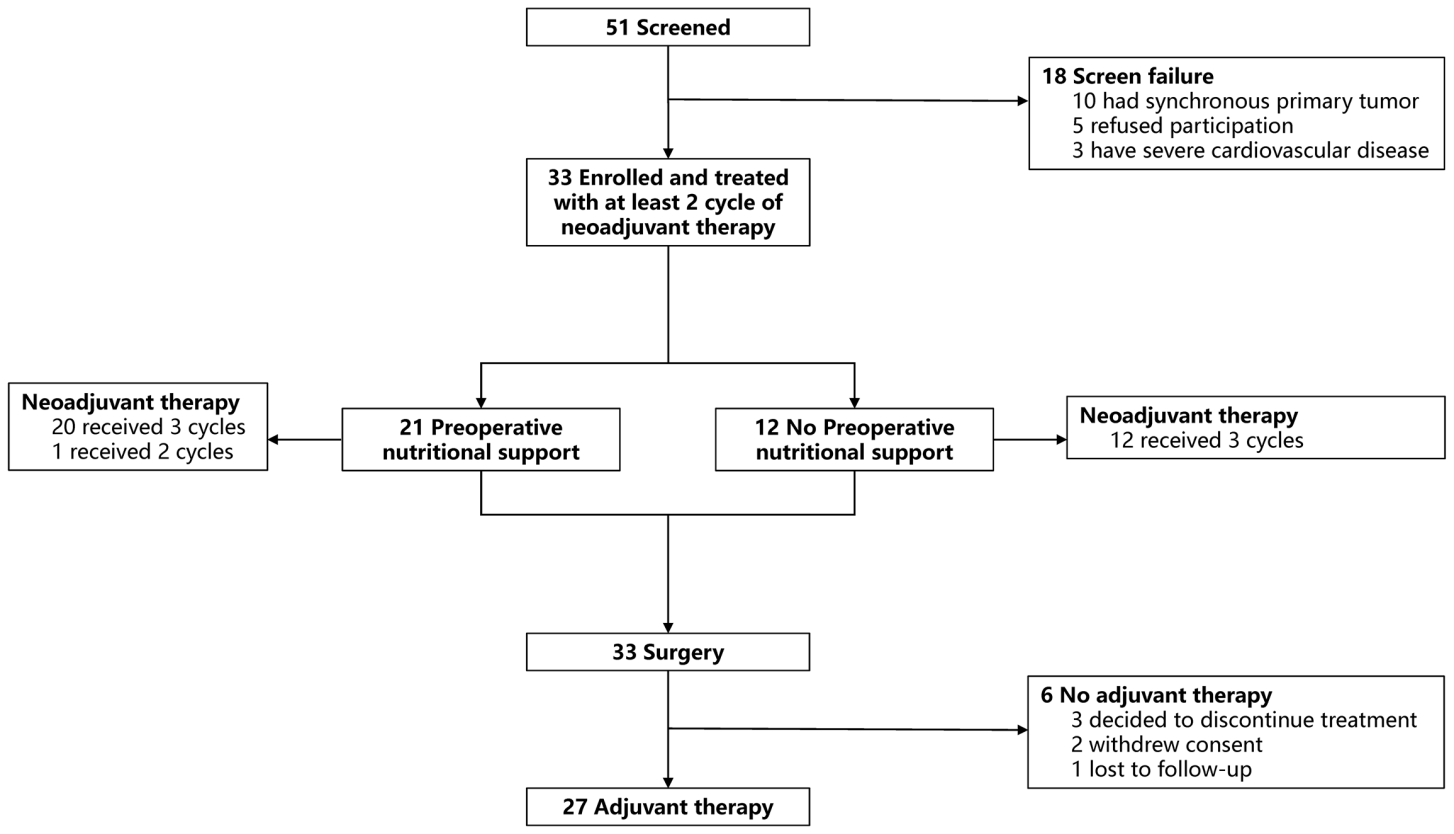
Patient enrollment and treatment process. The flowchart shows patient flow from screening to adjuvant treatment in the study: Fifty-one patients were screened, with nine excluded due to refusal or primary tumor reasons. Forty-two patients received at least one cycle of neoadjuvant therapy. Forty patients underwent surgery, among whom 25 received preoperative nutritional support and 15 did not. Thirty-two patients received postoperative adjuvant therapy.

Between September 2023 and December 2024, a total of 51 patients were screened at Tianjin Medical University Cancer Institute and Hospital. Of these, 33 patients met the eligibility criteria, received at least two dose of study treatment, and were included in the ITT population (**Figure 1**). Baseline demographic and clinical characteristics are summarized in **Table 1**. The median age was 60 years (range: 33-75), and 78.79% (26/33) of patients were male. Nearly all patients (93.94%, 31/33) were microsatellite stable (MSS). The majority presented a substantial tumor burden at enrollment, with 32 patients (96.97%) having cT4 disease and only 1 (3.03%) having cT3 disease. Only seven patients had negative lymph nodes, and all tumors were moderately to poorly differentiated. The median body mass index (BMI) was 22.32 kg/m², and 31 patients (93.94%) had a BMI ≥18.5 kg/m², indicating a generally preserved nutritional status. Six patients (18.18%) presented with gastric cancer-related symptoms at baseline, including pyloric obstruction and upper gastrointestinal bleeding.

**Table 1:** Baseline clinical characteristics of patients.

Characteristics	ITT	Surgery patients			
	All (n=33)	All (n=33)	PN (n=21)	Non-PN (n=12)	*P*-value
Age, years					
Median (range)	60 (33, 75)	60 (33, 75)	61 (33, 74)	53.50 (33, 75)	0.160
<65, n (%)	22 (66.67)	22 (66.67)	13 (61.90)	9 (75.00)	0.703
≥65, n (%)	11 (33.33)	11 (33.33)	8 (38.10)	3 (25.00)	
≥75, n (%)	1 (3.03)	1 (3.03)	0 (0.00)	1 (8.33)	
Sex, n (%)					1.000
Male	26 (78.79)	26 (78.79)	16 (76.19)	10 (83.33)	
Female	7 (21.21)	7 (21.21)	5 (23.81)	2 (16.67)	
BMI, kg/m²					
Median (range)	22.32 (17.17, 33.66)	22.32 (17.17, 33.66)	22.32 (19.57, 33.66)	22.61 (17.17, 28.76)	0.925
<18.5, n (%)	2 (6.06)	2 (6.06)	0 (0.00)	2 (16.67)	0.125
≥18.5, n (%)	31 (93.94)	31 (93.94)	21 (100.00)	10 (83.33)	
Gastric cancer-related symptoms at diagnosis, n (%)					
Yes	6 (18.18)	6 (18.18)	5 (23.81)	1 (8.33)	0.379
Pyloric obstruction	2 (6.06)	2 (6.06)	1 (4.76)	1 (8.33)	
Gastrointestinal bleeding	2 (6.06)	2 (6.06)	2 (9.52)	0 (0.00)	
Anemia	1 (3.03)	1 (3.03)	1 (4.76)	0 (0.00)	
Esophagitis	1 (3.03)	1 (3.03)	1 (4.76)	0 (0.00)	
Gastric retention	1 (3.03)	1 (3.03)	1 (4.76)	0 (0.00)	
Clinical stage, n (%)					
T stage					0.364
T3	1 (3.03)	1 (3.03)	0 (0.00)	1 (8.33)	
T4	32 (96.97)	32 (96.97)	21 (100.00)	11 (91.67)	
T4a	32 (96.97)	32 (96.97)	21 (100.00)	11 (91.67)	
N stage					1.000
N0	7 (21.21)	7 (21.21)	5 (23.81)	2 (16.67)	
N+	17 (51.52)	17 (51.52)	12 (57.14)	5 (41.67)	
N1	5 (15.15)	5 (15.15)	2 (9.52)	3 (25.00)	
N2	12 (36.36)	12 (36.36)	10 (47.62)	2 (16.67)	
Nx	9 (27.27)	9 (27.27)	4 (19.05)	5 (41.67)	
Signet-ring cell carcinoma, n (%)					0.271
Yes	4 (12.12)	4 (12.12)	4 (19.05)	0 (0.00)	
Histological type, n (%)					1.000
Intestinal	8 (24.24)	8 (24.24)	5 (23.81)	3 (25.00)	
Diffuse	8 (24.24)	8 (24.24)	4 (19.05)	4 (33.33)	
Mixed	7 (21.21)	7 (21.21)	4 (19.05)	3 (25.00)	
Not specified	10 (30.30)	10 (30.30)	8 (38.10)	2 (16.67)	
Differentiation degree, n (%)					0.535
Poor	28 (84.85)	28 (84.85)	17 (80.95)	11 (91.67)	
Moderate	3 (9.09)	3 (9.09)	3 (14.29)	0 (0.00)	
Not specified	2 (6.06)	2 (6.06)	1 (4.76)	1 (8.33)	
MSI status, n (%)					1.000
MSI-High	1 (3.03)	1 (3.03)	1 (4.76)	0 (0.00)	
MSS	31 (93.94)	31 (93.94)	19 (90.48)	12 (100.00)	
Not evaluated	1 (3.03)	1 (3.03)	1 (4.76)	0 (0.00)	
HER-2 status, n (%)					0.778
Negative					
IHC 0	18 (54.55)	18 (54.55)	12 (57.14)	6 (50.00)	
IHC 1+	11 (33.33)	11 (33.33)	6 (28.57)	5 (41.67)	
IHC 2+ with negative FISH	4 (12.12)	4 (12.12)	3 (14.29)	1 (8.33)	
PD-L1 CPS, n (%)					0.192
Negative (<1)	6 (18.18)	6 (18.18)	4 (19.05)	2 (16.67)	
Positive (≥1)	7 (21.21)	7 (21.21)	7 (33.33)	0 (0.00)	
1-5	2 (6.06)	2 (6.06)	2 (9.52)	0 (0.00)	
≥5	5 (15.15)	5 (15.15)	5 (23.81)	0 (0.00)	
Not evaluated	20 (60.61)	20 (60.61)	10 (47.62)	10 (83.33)	
EBV status					0.493
Negative	22 (66.67)	22 (66.67)	12 (57.14)	10 (83.33)	
Positive	2 (6.06)	2 (6.06)	2 (9.52)	0 (0.00)	
Not evaluated	9 (27.27)	9 (27.27)	7 (33.33)	2 (16.67)	

ITT, intention-to-treat.

Among the enrolled patients, 33 (100%) underwent curative-intent surgical resection, and 32 (96.97%) completed all three planned cycles of neoadjuvant therapy prior to surgery, and 21 (63.64%) received standardized PN support during the interval between completion of neoadjuvant therapy and surgery (**Table 2**). Baseline and treatment characteristics were generally comparable between patients who received PN and those who did not (**Tables 1**, **2**). All patients achieved R0 resection. Twenty-eight patients underwent laparoscopic resection with or without robotic assistance, and the remaining 5 underwent open surgery. The mean estimated intraoperative blood loss was 220.30 mL, and no patient required intraoperative transfusion. No perioperative deaths were reported. As of the data cutoff (June 1, 2025), the median follow-up duration of all patients was 12.13 months (range: 2.63-20.30). Twenty-seven patients received protocol-defined adjuvant therapy postoperatively, with a median of three cycles administered (range: 1-7).

**Table 2:** Treatment characteristics of PN group and non-PN group.

Characteristics	All (n=33)	PN (n=21)	Non-PN (n=12)	*P*-value
Neoadjuvant therapy, n (%)				1.000
Completed all 3 planned cycles	32 (96.97)	20 (95.24)	12 (100.00)	
Other cycles	1 (3.03)	1 (4.76)	0 (0.00)	
Received 1–2 cycles	1 (3.03)	1 (4.76)	0 (0.00)	
Received >3 cycles	0 (0.00)	0 (0.00)	0 (0.00)	
Preoperative PN support, n (%)				/
Yes	21 (63.64)	21 (100.00)	0 (0.00)	
No	12 (36.36)	0 (0.00)	12 (100.00)	
Interval between final neoadjuvant dose and surgery, days				0.022
Median (range)	38.00 (20.00, 75.00)	42.00 (24.00, 75.00)	32.00 (20.00, 42.00)	
Preoperative weight loss, n (%)				0.469
Yes	18 (54.55)	10 (47.62)	8 (66.67)	
<1 kg	0 (0.00)	0 (0.00)	0 (0.00)	
1–2 kg	2 (6.06)	1 (4.76)	1 (8.33)	
2–3 kg	5 (15.15)	3 (14.29)	2 (16.67)	
>3 kg	11 (33.33)	6 (28.57)	5 (41.67)	
Surgical approach, n (%)				0.328
Laparoscopic	28 (84.85)	19 (90.48)	9 (75.00)	
Robotic-assisted	4 (12.12)	4 (19.05)	0 (0.00)	
Open	5 (15.15)	2 (9.52)	3 (25.00)	
Surgical procedure, n (%)				0.282
Total gastrectomy	17 (51.52)	9 (42.86)	8 (66.67)	
Subtotal gastrectomy	16 (48.48)	12 (57.14)	4 (33.33)	
Intraoperative blood loss, mL				0.068
mean ± S.D.	220.30 ± 222.65	146.19 ± 89.19	350.00 ± 317.66	
R0 resection, n (%)				1.000
Yes	33 (100.00)	21 (100.00)	12 (100.00)	
Adjuvant therapy				1.000
Yes, n (%)	27 (81.82)	17 (80.95)	10 (83.33)	
Cycles, Median (range)	3 (1, 7)	4 (1, 7)	3 (1, 6)	
EFS events observed, n (%)	6 (18.18)	4 (19.05)	2 (16.67)	1.000
Duration of follow-up, months				
Median (range)	12.13 (2.63, 20.30)	12.13 (2.63, 18.00)	12.07 (5.37, 20.30)	

PN, received parenteral nutrition; non-PN, not received parenteral nutrition; EFS, event-free survival.

**Figure 2:**
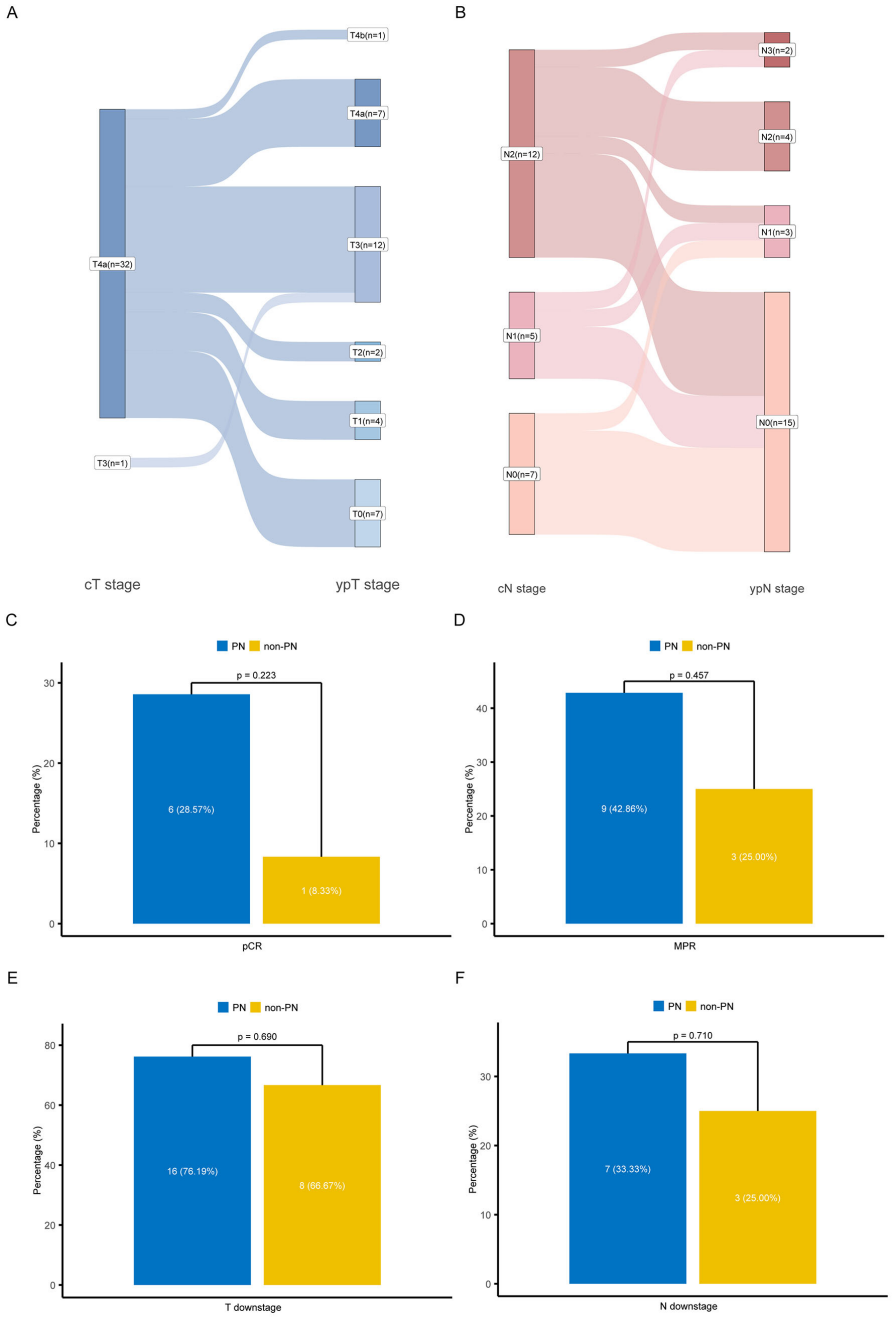
Staging changes in patients after neoadjuvant therapy and the effect of parenteral nutrition on pathological responses. **(A, B)** Sankey diagram of changes in T and N stage before and after neoadjuvant therapy. Streamlines present the distribution of patients with different stages converting to each stage after treatment. **(C-F)** Comparison of pathological response in PN (blue) and non-PN (yellow). **(C)** Comparison the pCR of PN group and non-PN group; **(D)** Comparison the MPR of PN group and non-PN group; **(E)** Comparison of T downstage in PN group and non-PN group; **(F)** Comparison of N downstage in PN group and non-PN group. PN, received parenteral nutrition; non-PN, not received parenteral nutrition; pCR, pathological complete response; MPR, major pathological response.

Among all the patients who underwent surgery, the pCR and MPR rates were 21.21% (95% CI: 8.98-38.91) and 36.36% (95% CI: 20.40-54.88), respectively. Twenty-four patients experienced pathological downstaging in the T stage (72.73%), and 10 had downstaging in the N stage (30.30%) (**Figures 2A, B**). No significant differences were observed in pCR, MPR, or downstaging rates between patients with or without PN support (**Figures 2C, D**; all *p*-values >0.05). A subgroup analysis was conducted to explore baseline clinicopathologic factors potentially associated with achieving MPR (**Table 3**). Patients who achieved MPR were more likely to have moderately differentiated tumors compared to those who did not (25.0% vs. 0.0%, *p* = 0.027). No significant differences were observed between MPR and non-MPR patients in terms of gastric cancer-related symptoms, presence of signet-ring cell carcinoma, T or N stage, PD-L1 expression, or HER2 expression.

**Table 3:** Baseline clinicopathology in patients achieving MPR and non-MPR.

Characteristics	MPR (n=12)	Non-MPR (n=21)	*P*-value
Age, years			0.696
Median (range)	60.50 (33, 75)	60 (33, 73)	1.000
<65	8 (66.67)	14 (66.67)	
≥65	4 (33.33)	7 (33.33)	
Sex, n (%)			0.686
Male	9 (75.00)	17 (80.95)	
Female	3 (25.00)	4 (19.05)	
BMI, kg/m²			
Median (range)	22.37 (19.57, 33.66)	22.32 (17.17, 28.34)	0.537
<18.5	0 (0.00)	2 (9.52)	0.523
≥18.5	12 (100.00)	19 (90.48)	
Gastric cancer-related symptoms at diagnosis, n (%)			0.643
Yes	3 (25.00)	3 (14.29)	
Clinical stage, n (%)			
T stage			1.000
T3	0 (0.00)	1 (4.76)	
T4	12 (100.00)	20 (95.24)	
N stage			0.356
N0	4 (33.33)	3 (14.29)	
N+	5 (41.67)	12 (57.14)	
Signet-ring cell carcinoma, n (%)			1.000
Yes	1 (8.33)	3 (14.29)	
Histological type, n (%)			1.000
Intestinal	2 (16.67)	6 (28.57)	
Diffuse	2 (16.67)	6 (28.57)	
Mixed	1 (8.33)	6 (28.57)	
Differentiation degree, n (%)			0.027
Poor	7 (58.33)	21 (100.00)	
Moderate	3 (25.00)	0 (0.00)	
MSI status, n (%)			1.000
MSI-High	0 (0.00)	1 (4.76)	
MSS	11 (91.67)	20 (95.24)	
HER-2 status, n (%)			0.300
Negative			
IHC 0	5 (41.67)	13 (61.90)	
IHC 1+ or 2+ with negative FISH	7 (58.33)	8 (38.10)	
PD-L1 CPS, n (%)			1.000
Negative (<1)	1 (8.33)	5 (23.81)	
Positive (≥1)	2 (16.67)	5 (23.81)	
EBV status			1.000
Negative	5 (41.67)	17 (80.95)	
Positive	0 (0.00)	2 (9.52)	

MPR, major pathological response; EBV, Epstein Barr virus.

**Figure 3:**
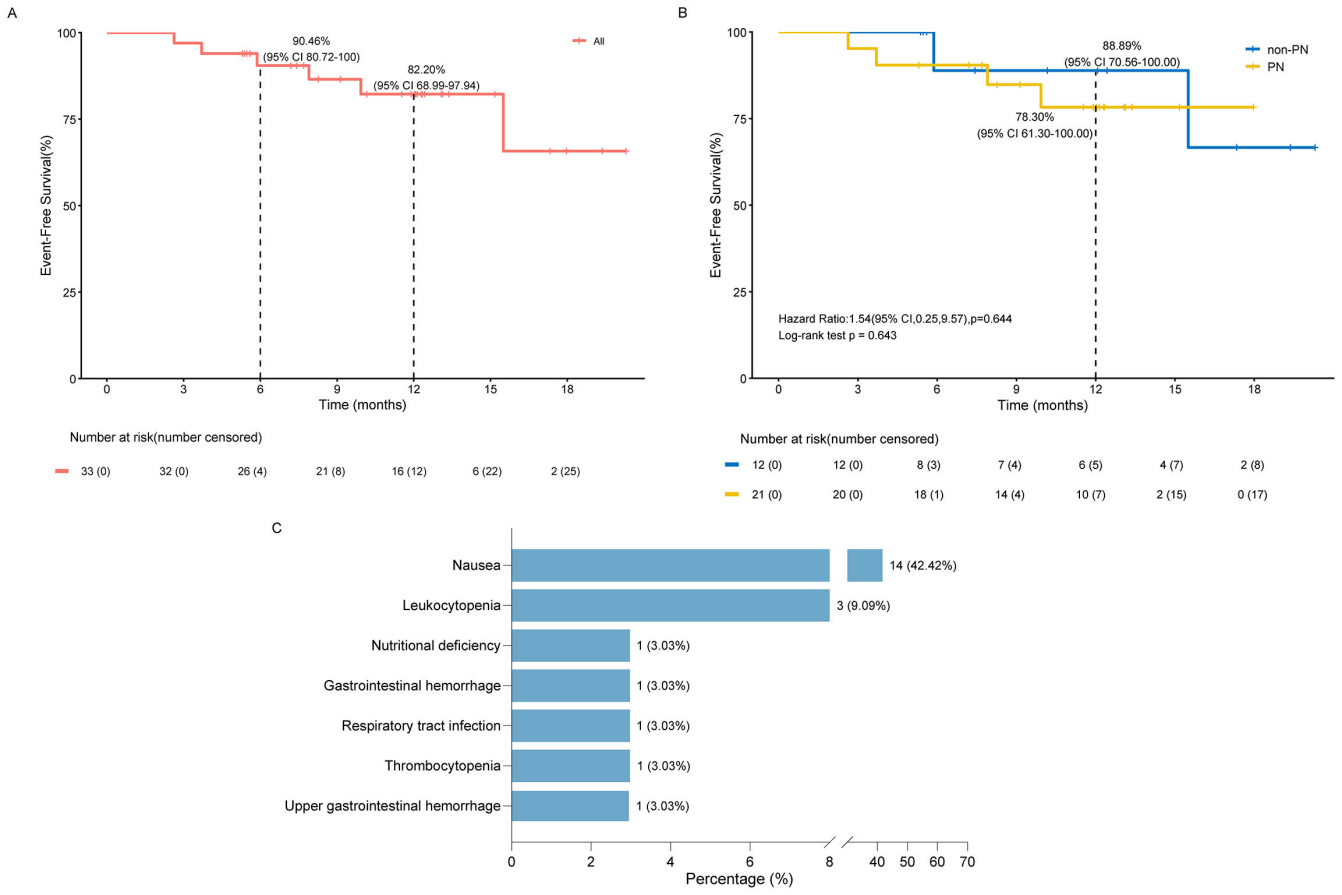
Analysis of EFS and treatment safety. **(A)** The EFS curve for all patients; **(B)** Comparison the EFS curves of PN group and non-PN group; **(C)** Distribution of AEs during neoadjuvant therapy. PN, received parenteral nutrition; non-PN, not received parenteral nutrition; EFS, event-free survival.

At the data cutoff, six patients (18.18%) experienced EFS events. The median EFS was not reached, and the 12-month EFS rate was 82.20% (95% CI: 68.99-97.94) (**Figure 3A**). Among patients who achieved MPR, only one patient who declined adjuvant therapy experienced an EFS event more than one year after surgery and was undergoing systemic therapy for recurrence. Twelve-month EFS rates were 78.30% (95% CI: 61.30-100.00) in the PN group and 88.89% (95% CI: 70.56-100.00) in the non-PN group, with no statistically significant difference (**Figure 3B**; log-rank *p* = 0.643). No deaths were reported during the follow-up period. OS data remains immature.

Patients generally tolerated the study treatment well. During neoadjuvant therapy, 15 patients (45.45%) experienced mild adverse events (AEs), with no grade ≥3 AEs reported. The most common AEs included nausea, decreased white blood cell count, and malnutrition (**Figure 3C**).

**Figure 4:**
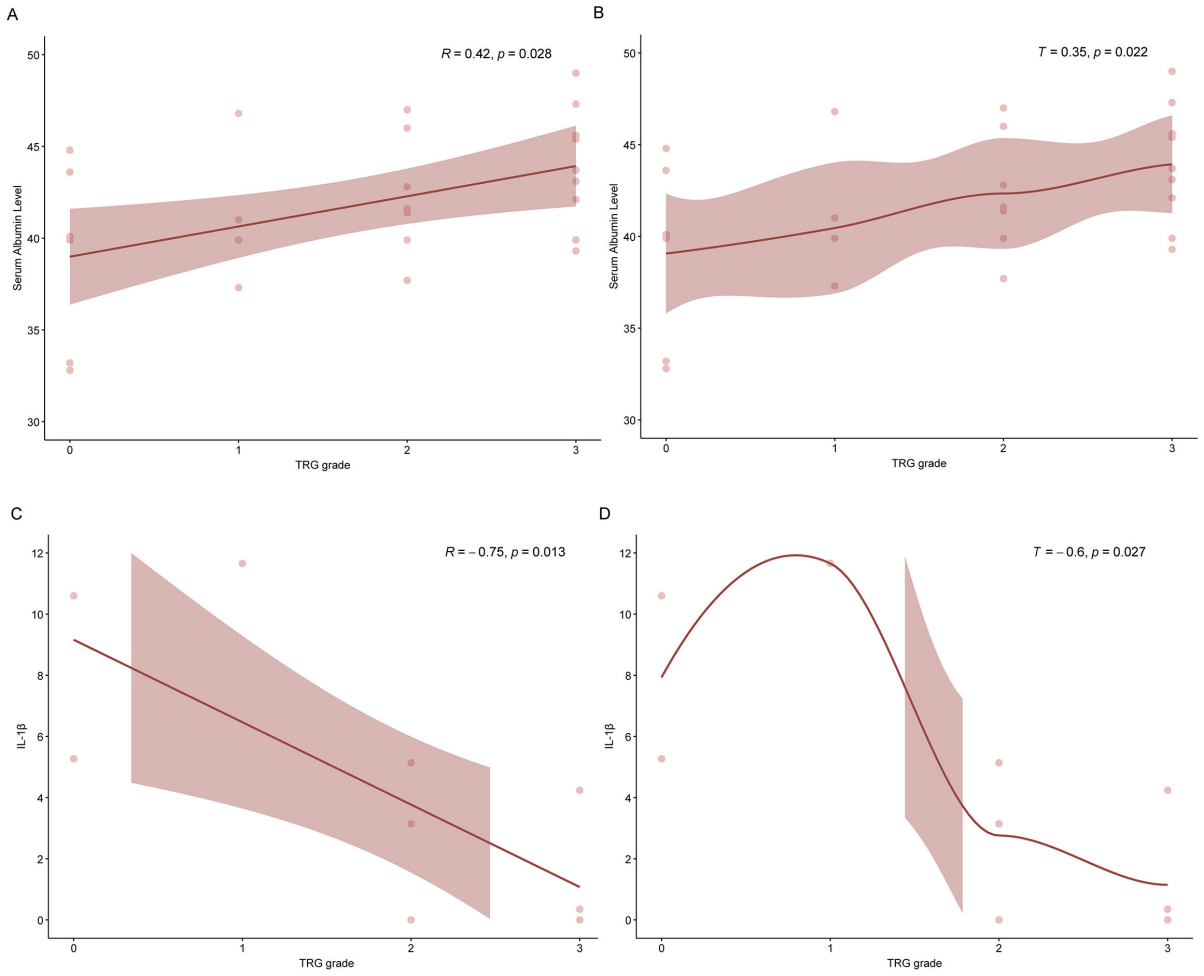
Correlation analyses of immune markers with TRG based on preoperative peripheral blood samples: **(A)** Spearman’s correlation between serum albumin level and TRG; **(B)** Kendall’s correlation between serum albumin level and TRG; **(C)** Spearman’s correlation between IL-1β and TRG; **(D)** Kendall’s correlation between IL-1β and TRG. IL-1β, interleukin-1β; TNF-α, tumor necrosis factor-α; TRG, tumor regression grade.

To investigate the potential predictive value of immune and inflammatory markers, we conducted correlation analyses between peripheral blood-based biomarkers and TRG. Both baseline and preoperative (post-neoadjuvant, pre-surgical) blood samples were included in this exploratory assessment. Correlation analyses between peripheral blood-based immune markers and TRG were conducted using non-parametric methods (Spearman’s ρ and Kendall’s τ-b), based on both baseline and preoperative blood samples (**Supplementary Table S1**, 2, **Figures 4A-D**). A notable correlation was observed between baseline serum albumin level (g/L) and TRG (ρ = 0.42, p = 0.028; τ = 0.35, p = 0.022) (**Figures 4A, B**). Additionally, analysis of preoperative biomarkers revealed a significant negative correlation between interleukin-1β (IL-1β) and TRG (ρ = –0.75, p = 0.013; τ = –0.60, p = 0.027) (**Figures 4C, D**), indicating that higher preoperative IL-1β levels tended to be associated with better tumor regression. Other markers, including IL-4, IL-6, interferon-γ (IFN-γ), T-cell subsets, tumor necrosis factor-α (TNF-α), albumin, neutrophil and lymphocyte counts, and derived indices such as neutrophil-to-lymphocyte ratio (NLR), platelet-to-lymphocyte ratio (PLR), systemic immune-inflammation index (SII), and prognostic nutritional index (PNI), showed no statistically significant correlations with TRG at either time point.

To evaluate the potential effects of preoperative PN support on systemic immune status, we compared peripheral immune and hematologic biomarkers between patients who received PN and those who did not at three distinct timepoints: baseline, pre-surgery, and post-surgery. At the baseline assessment, the levels of cytokines, including IL-4, IL-6, IFN-γ, and TNF-α, as well as the distribution of lymphocyte subsets (e.g., CD3^+^, CD4^+^, CD8^+^ T cells), neutrophil and lymphocyte counts, serum albumin, and composite indices such as the NLR, PLR, SII, and PNI, showed no statistically significant differences between the two groups (**Supplementary Table S3**). These results indicate a comparable immunonutritional status prior to PN intervention. By the preoperative timepoint, the PN group exhibited a significantly higher lymphocyte count (1.68 ± 0.49 × 10^9^/L vs. 1.29 ± 0.38 × 10^9^/L, p = 0.024) and a markedly lower PLR (95.88 ± 30.01 vs. 137.57 ± 48.83, p = 0.005) compared with the non-PN group (**Supplementary Table S4**). These results indicate that short-term PN support was associated with preservation of lymphocyte levels and a reduction in systemic inflammatory burden during the neoadjuvant treatment period. At the postoperative evaluation, both groups exhibited expected changes associated with surgical stress. However, there were no significant differences between the PN and non-PN groups in terms of cytokine expression, immune cell populations, or inflammatory and nutritional indices. Notably, postoperative values of serum albumin, lymphocyte counts, NLR, SII, PLR, and PNI remained statistically comparable between groups (**Supplementary Table S5**). Collectively, these findings suggest that perioperative PN support, as implemented in this cohort, may confer short-term benefits in maintaining lymphocyte levels and modulating systemic inflammation during the neoadjuvant phase, but does not appear to significantly influence postoperative immune recovery or nutritional status.

**Figure 5:**
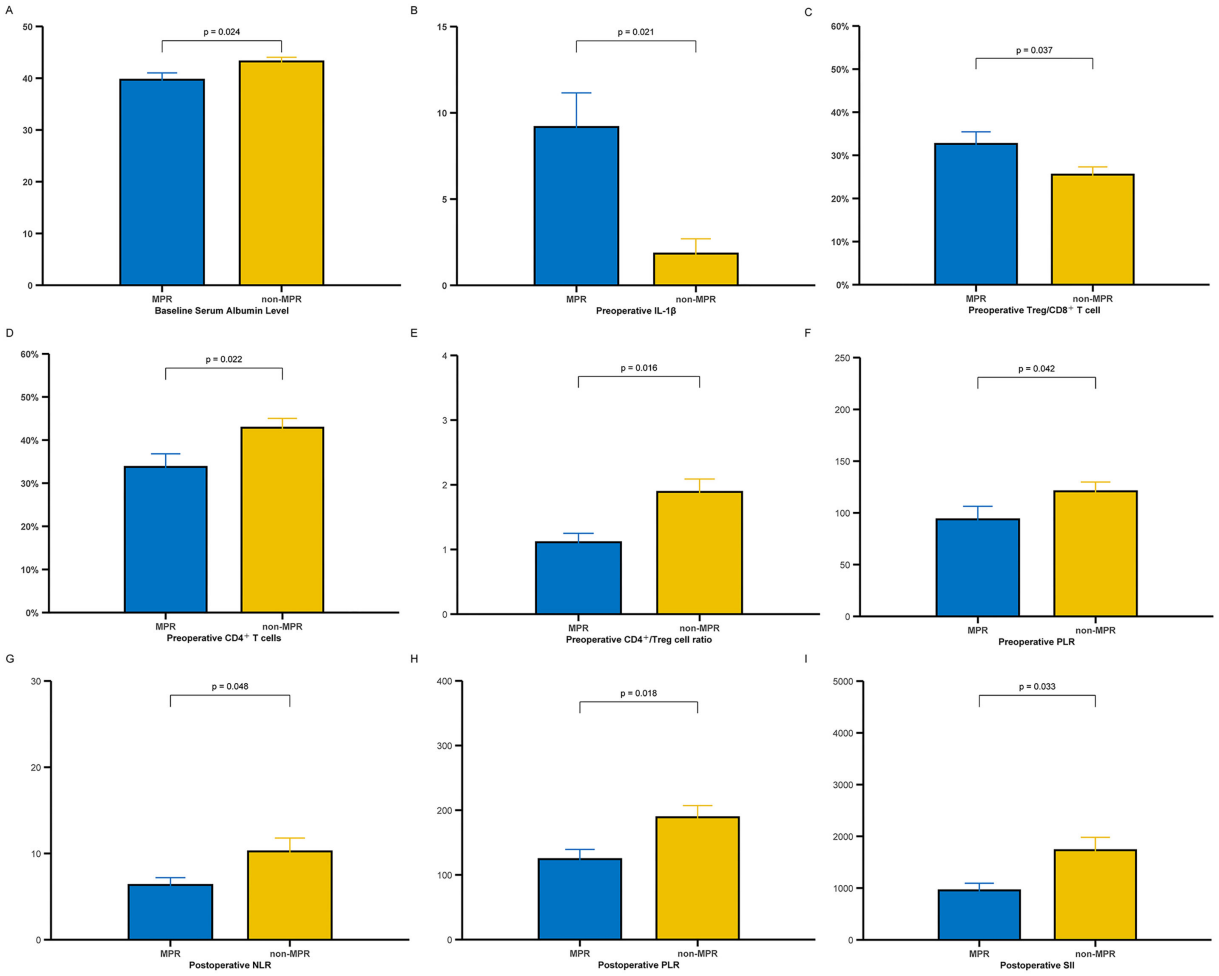
Comparison of immune markers between MPR and non-MPR patients on preoperative peripheral blood samples: **(A)** Baseline serum albumin level; **(B)** Preoperative serum IL-1β level; **(C)** The preoperative Treg/CD8^+^ ratio; **(D)** The preoperative CD4^+^ T cells level; **(E)** The preoperative CD4^+^/Treg ratio; **(F)** The preoperative PLR; **(G)** The postoperative NLR; **(H)** The postoperative PLR; **(I)** The postoperative SII. MPR, major pathological response; PLR, platelet-to-lymphocyte ratio; NLR, neutrophil-to-lymphocyte ratio; IL-1β, interleukin-1β; SII, systemic immune-inflammation index.

To explore whether systemic immune status was associated with pathological response, we compared a panel of circulating inflammatory and immune-related parameters between patients who achieved MPR and those who did not (non-MPR) at three perioperative time points: baseline, preoperative, and postoperative. At baseline, Some several immune markers, including the levels of cytokines such as IL-4, IL-6, and IFN-γ, as well as T and B lymphocyte subsets, showed numerical trends favoring the MPR group. Notably, MPR patients had numerically lower NLR, PLR, and SII, suggesting a relatively favorable immune milieu (**Supplementary Table S6**). But serum albumin level was significantly lower in the MPR group than in the non-MPR group (39.7 ± 4.4 vs. 43.2 ± 3.3 g/L, *P* = 0.024), which may be attributed to multiple factors such as hepatic function and tumor-related metabolic consumption (**Figure 5A**). Before surgery, several immune parameters diverged further between the two groups (**Supplementary Table S7**). IL-1β was significantly elevated in the MPR group (9.18 ± 3.42 vs. 1.84 ± 2.26, *P* = 0.021), potentially indicating a more active proinflammatory state associated with effective tumor clearance (**Figure 5B**). Specifically, patients who achieved MPR exhibited a higher Treg/CD8^+^ T cell (32.64 ± 8.43% vs. 25.52 ± 7.63%, *P* = 0.037) but lower proportions of helper/inducer T cells (CD4^+^ T cells) (33.76 ± 9.15% vs. 42.87 ± 9.09%, *P* = 0.022) (**Figures 5C, D**). Consequently, the CD4^+^/Treg cell ratio was significantly lower in the MPR group (1.11 ± 0.42 vs. 1.89 ± 0.85, *P* = 0.016) (**Figure 5E**), suggesting an altered balance of helper and suppressor T cell subsets in patients who responded well to neoadjuvant therapy, and the MPR group exhibited a significantly lower PLR value (93.74 ± 41.95 vs. 120.83 ± 40.92, *P* = 0.042) (**Figure 5F**), consistent with a less suppressive immune state prior to surgery. Notably, the difference in serum albumin levels observed at baseline disappeared prior to surgery, implying that systemic inflammation and metabolic disturbances were ameliorated after treatment. Collectively, these changes suggest a shift toward an activated and less immunosuppressive systemic profile among MPR patients prior to surgery. Postoperatively, the intergroup differences in immune indices appeared attenuated, and no statistically significant differences were observed in cytokine levels, lymphocyte subsets. However, several systemic inflammation markers remained lower in MPR patients, including NLR (6.35 ± 2.93 vs. 10.24 ± 7.12, *P* = 0.048), PLR (124.29 ± 51.90 vs. 188.94 ± 83.35, *P* = 0.018), and SII (955.71 ± 478.03 vs. 1,731.93 ± 1,141.28, *P* = 0.033) (**Figures 5G–I**). These findings suggest a persistently attenuated systemic inflammatory response and a more balanced immune profile in patients who achieved MPR after neoadjuvant immunochemotherapy. Taken together, systemic immune remodeling during neoadjuvant immunotherapy may influence pathological response. In particular, preoperative cytokine expression and T-cell composition appeared to correlate with MPR, highlighting the potential utility of these markers as predictive indicators for treatment efficacy.

## 4 Discussion

This prospective study is the first to evaluate a perioperative treatment strategy combining serplulimab with SOX chemotherapy in patients with locally advanced gastric cancer. Uniquely, it also explores the immunologic impact of perioperative PN within the context of neoadjuvant immunotherapy. Despite the inclusion of patients with a high baseline tumor burden, with over 95% presenting with cT4 disease, the combination regimen achieved a promising pCR rate of 21.21% and an MPR rate of 36.36% after three cycles of serplulimab-based neoadjuvant treatment. And, the 12-month EFS rate reached 82.20% (95% CI: 68.99-97.94). Importantly, this study provides novel insights into systemic immune remodeling during neoadjuvant immunotherapy, showing that preoperative shifts in circulating cytokine profiles (e.g., IL-1β, TNF-α) and T-cell subset composition (e.g., CD4^+^/Treg ratio, Treg/CD8^+^ ratio) may correlate with improved pathological response. In addition, our data suggest that short-term perioperative PN may confer transient benefits by preserving lymphocyte counts and mitigating systemic inflammatory burden during the neoadjuvant treatment phase, although it did not significantly affect postoperative immune recovery or nutritional status in this cohort. Collectively, these findings underscore the feasibility and therapeutic potential of serplulimab-based perioperative treatment and highlight the value of non-invasive blood-based immune markers as predictive tools for response. They also suggest new opportunities to optimize perioperative immunonutritional strategies in the management of gastric cancer.

This study’s observed pCR rate of 21.21% and 12-month EFS rate of 82.20% are relatively modest compared to those seen in other pivotal trials investigating neoadjuvant immunotherapy in gastric cancer. For example, the GERCOR NEONIPIGA trial, a multicenter phase II study conducted in France, assessed the combination of nivolumab and ipilimumab as neoadjuvant treatment for MSI-H/dMMR gastric or gastroesophageal junction adenocarcinomas. This trial achieved a 58.6% pCR rate among the 29 patients who underwent surgery, with all patients receiving R0 resection, suggesting the potential of this treatment regimen in this specific population (20). In addition, the NEOSUMMIT-01 trial, a randomized phase II study in China, evaluated the addition of the PD-1 inhibitor toripalimab to chemotherapy for cT3-4aN+M0 resectable gastric cancer. This trial showed significant improvements in both the TRG 0/1 rate (44.4% vs. 20.4%) and pCR rate (22.2% vs. 7.4%) with the addition of PD-1 inhibition, demonstrating promising results when combining immunotherapy with chemotherapy in this cohort (21). The differences in pCR and EFS between these studies and our findings could be attributed to factors such as patient heterogeneity and the relatively small sample size of our study. However, when compared to larger phase III trials, our results align with broader trends observed in studies like KEYNOTE-585, which demonstrated a pCR rate of 12.9% and a median EFS of 44.4 months in patients receiving neoadjuvant pembrolizumab combined with chemotherapy (12). Similarly, the MATTERHORN trial reported a pCR rate of 19% and median EFS not reached in patients treated with durvalumab plus FLOT chemotherapy (13). Notably, our treatment strategy involving perioperative serplulimab (HLX10) combined with SOX chemotherapy mirrors the ongoing phase III registrational trial NCT04139135, a randomized, double-blinded, multicenter study designed to formally evaluate the efficacy and safety of this regimen compared to placebo plus chemotherapy. In NCT04139135, patients receive three cycles of neoadjuvant HLX10 plus SOX chemotherapy, followed by surgery and up to 12 months of adjuvant HLX10 monotherapy, with EFS assessed by an independent radiologic review committee as the primary endpoint. However, the NCT04139135 trial exclusively enrolls patients with PD-L1 CPS ≥5, whereas our study broadened inclusion criteria to encompass all patients irrespective of PD-L1 status, and our subgroup analysis revealed no significant association between PD-L1 expression and achievement of MPR. Given the alignment between the two trials’ treatment strategies, our exploratory findings provide important preliminary evidence supporting the rationale and feasibility of this perioperative regimen, underscoring the necessity of further validation from this ongoing phase III trial. Collectively, our study reinforces the potential value of neoadjuvant immunotherapy for LAGC, while highlighting the need for ongoing refinement of patient selection, biomarker identification, and therapeutic optimization to fully realize clinical benefits.

Neoadjuvant immunotherapy has demonstrated significant promise in reshaping the immune microenvironment, influencing tumor progression and response to therapy (22). In our study, preoperative immune signatures, including cytokine levels such as IL-1β and TNF-α, as well as changes in T-cell subset ratios, were shown to correlate with pathological response. Specifically, patients who achieved MPR exhibited higher IL-1β levels, indicating an active proinflammatory immune state, along with a more favorable balance of CD4^+^/Treg cells, an elevated Treg/CD8^+^ ratio, and a lower PLR. This suggests that the systemic immune response induced by neoadjuvant therapy plays a pivotal role in driving tumor regression and enhancing treatment efficacy. Our findings regarding systemic immune remodeling are further supported by emerging multi-omics evidence from other neoadjuvant immunotherapy trials. A phase II study (NCT03878472) evaluating camrelizumab combined with apatinib and chemotherapy in cT4a/bN^+^ gastric cancer, complete and major pathological response rates were 15.8% and 26.3%, respectively, which are comparable to our observed rates. Notably, multi-omics profiling in that study revealed dynamic alterations in the tumor microenvironment (TME), including reductions in Treg cells and increases in CD8^+^T cells, dendritic cells, T helper cells, and M1 macrophages in patients who achieved pathological response. Additionally, transcriptomic analysis identified distinct baseline immune signatures, such as elevated expression of immune checkpoint molecules (CD274, CTLA4), cytolytic genes (GZMB, PRF1), and enhanced IFN-γ and T cell exhaustion signatures in responders. These changes were accompanied by shifts in TME phenotypes from immunologically “cold” (depleted or fibrotic) to “immune-enriched” profiles following therapy (23). In addition, a recent meta-analysis focusing on advanced gastric cancer patients in the era of immunotherapy reported that elevated PLR was significantly associated with worse overall survival (OS), with a pooled hazard ratio (HR) of 1.77 (95% CI: 1.44–2.17, P < 0.00001), and shorter progression-free survival (PFS), with a pooled HR of 1.61 (95% CI: 1.33–1.96, P < 0.00001). This adverse prognostic effect of high PLR was consistently observed across all subgroups, with no significant heterogeneity (24). Collectively, these findings underscore that integrating the immunophenotypic features of the local tumor microenvironment with systemic immune−inflammatory biomarkers is essential for a comprehensive understanding of the mechanisms driving response to neoadjuvant immunotherapy in gastric cancer.

Our investigation into the role of standardized perioperative PN in modulating systemic immune status revealed a transient preservation of lymphocyte counts and a modest reduction in systemic inflammation during the neoadjuvant treatment phase. Nutrition-related immune modulation is widely recognized as an important component of surgical recovery and treatment tolerance (25), Previous studies have shown that malnutrition can lead to immune dysfunction and adversely affect oncologic outcomes (15, 16). However, our data suggest that short-term, standardized preoperative PN alone may be insufficient to meaningfully alter the systemic immune landscape during immunotherapy, as postoperative assessments revealed no significant differences between the PN and non-PN groups in circulating cytokines, immune cell populations, or inflammatory and nutritional markers. These findings highlight the need for further research incorporating more refined immune monitoring techniques and more individualized nutritional strategies to better understand the interplay between nutrition, systemic immunity, and tumor response. Taken together, our results underscore the clinical value of immune profiling as a complementary tool to improve the precision of neoadjuvant immunotherapy for gastric cancer. By integrating systemic immune signatures with tumor characteristics, clinicians may enhance the accuracy of treatment response prediction and develop more personalized perioperative strategies for patients with locally advanced disease.

This study has several limitations that should be acknowledged. First, its single-arm, single-center design and relatively small sample size limit the generalizability of the findings and increase the risk of selection bias. Second, although immunologic profiling was conducted at multiple perioperative timepoints, the scope of immune assessment was confined to circulating cytokines and lymphocyte subsets. Broader immune analyses, including functional immune assays and tumor microenvironment profiling, are warranted to provide more mechanistic insights. Third, while we explored the impact of perioperative PN on systemic immunity, the exploratory nature of this study and limited sample size preclude definitive conclusions regarding its clinical or immunologic effects. Finally, the relatively short follow-up duration limits our ability to assess long-term survival endpoints, such as overall survival, and extended follow-up is needed to confirm the prognostic relevance of the immune and pathological responses observed.

## 5 Conclusion

This prospective study is the first to report a perioperative treatment strategy combining serplulimab with SOX chemotherapy in patients with locally advanced gastric cancer, while also exploring the immunologic implications of perioperative parenteral nutrition. Despite the inclusion of patients with high baseline tumor burden, the regimen achieved encouraging pathological responses, with a pCR rate of 21.21% and a 12-month EFS rate of 82.20%. Dynamic changes in preoperative cytokine expression and T-cell subset ratios were associated with pathological response, suggesting that systemic immune remodeling contributes to therapeutic efficacy. While perioperative PN appeared safe and immunologically neutral, its direct impact on immune modulation was limited in this setting. Collectively, these findings support the feasibility of serplulimab-based neoadjuvant immunotherapy in gastric cancer and highlight the potential of peripheral immune biomarkers as predictive tools. The ongoing phase III trial (NCT04139135) will validate the efficacy of this serplulimab-based perioperative immunochemotherapy strategy in LAGC, and further mechanistic investigations are warranted to deepen our understanding of the immune-tumor-nutrition axis in the neoadjuvant setting.

## Data Availability

The original contributions presented in the study are included in the article/**Supplementary Material**. Further inquiries can be directed to the corresponding authors.
