# Supplementary material for: Neoadjuvant serplulimab and SOX chemotherapy for locally advanced gastric cancer: pathological responses and systemic immune signatures from a phase II trial
Source: Front Immunol. 2026 Jan 12;16:1702737. doi: 10.3389/fimmu.2025.1702737 (PMC12832876; doi:10.3389/fimmu.2025.1702737)
Supplement: Supplementary file 1 [file Table1.docx]

Supplementary Material

# Supplementary Tables

**Table S1. Correlation of baseline hematologic markers with TRG.**

| Haematological indicators | n | Rho (ρ) | p | tau-b (τ) | p |
| --- | --- | --- | --- | --- | --- |
| IL-1β | 1 | - | - | - | - |
| IL-4 | 18 | 0.16 | 0.528 | 0.11 | 0.575 |
| IL-6 | 18 | 0.17 | 0.512 | 0.14 | 0.473 |
| IFN-γ | 18 | 0.20 | 0.434 | 0.13 | 0.497 |
| TNF-α | 18 | 0.31 | 0.204 | 0.23 | 0.216 |
| Average Lymphocyte Count (/μL) | 18 | -0.36 | 0.145 | -0.26 | 0.166 |
| B Cells (%) | 18 | -0.32 | 0.194 | -0.26 | 0.166 |
| T Cells (%) | 18 | 0.34 | 0.165 | 0.23 | 0.220 |
| Treg/CD8⁺ T cell (%) | 18 | 0.01 | 0.970 | -0.07 | 0.722 |
| CD4⁺ T cells (%) | 18 | 0.02 | 0.943 | 0.01 | 0.968 |
| CD4⁺/Treg cell ratio | 18 | 0.13 | 0.608 | 0.16 | 0.406 |
| NK Cells (%) | 18 | -0.09 | 0.723 | -0.07 | 0.722 |
| Serum Albumin Level (g/L) | 27 | 0.42 | 0.028 | 0.35 | 0.022 |
| Neutrophil Count (×10⁹/L) | 28 | 0.06 | 0.778 | 0.05 | 0.726 |
| Lymphocyte Count (×10⁹/L) | 28 | -0.13 | 0.517 | -0.08 | 0.564 |
| Platelet Count (×10⁹/L) | 28 | 0.23 | 0.236 | 0.19 | 0.195 |
| PNI | 27 | 0.29 | 0.139 | 0.24 | 0.113 |
| NLR | 28 | 0.21 | 0.286 | 0.17 | 0.258 |
| PLR | 28 | 0.19 | 0.345 | 0.12 | 0.399 |
| SII | 28 | 0.20 | 0.318 | 0.15 | 0.313 |

IL-1β, interleukin-1β; IL-4, interleukin-4; IL-6, interleukin-6; IFN-γ, interferon-γ; TNF-α, tumor necrosis factor-α; NK Cells, Natural Killer Cells; PNI, prognostic nutritional index; NLR, neutrophil-to-lymphocyte ratio; PLR, platelet-to-lymphocyte ratio; SII, systemic immune-inflammation index.

**Table S2. Correlation of preoperative hematologic markers with TRG.**

| Haematological indicators | n | Rho (ρ) | p | tau-b (τ) | p |
| --- | --- | --- | --- | --- | --- |
| IL-1β | 10 | -0.75 | 0.013 | -0.60 | 0.027 |
| IL-4 | 26 | -0.04 | 0.842 | -0.03 | 0.852 |
| IL-6 | 26 | 0.11 | 0.592 | 0.07 | 0.657 |
| IFN-γ | 26 | 0.13 | 0.540 | 0.09 | 0.543 |
| TNF-α | 26 | 0.33 | 0.102 | 0.25 | 0.105 |
| Average Lymphocyte Count (/μL) | 27 | -0.13 | 0.530 | -0.09 | 0.553 |
| B Cells (%) | 27 | 0.15 | 0.457 | 0.10 | 0.495 |
| T Cells (%) | 27 | 0.07 | 0.740 | 0.06 | 0.708 |
| Treg/CD8⁺ T cell (%) | 27 | -0.30 | 0.130 | -0.24 | 0.118 |
| CD4⁺ T cells (%) | 27 | 0.18 | 0.370 | 0.14 | 0.344 |
| CD4⁺/Treg cell ratio | 27 | 0.33 | 0.090 | 0.25 | 0.094 |
| NK Cells (%) | 27 | -0.05 | 0.798 | -0.03 | 0.843 |
| Serum Albumin Level (g/L) | 32 | 0.09 | 0.639 | 0.05 | 0.708 |
| Neutrophil Count (×10⁹/L) | 32 | 0.15 | 0.412 | 0.11 | 0.414 |
| Lymphocyte Count (×10⁹/L) | 32 | -0.16 | 0.384 | -0.11 | 0.434 |
| Platelet Count (×10⁹/L) | 32 | 0.09 | 0.639 | 0.05 | 0.695 |
| PNI | 32 | -0.05 | 0.790 | -0.08 | 0.563 |
| NLR | 32 | 0.24 | 0.186 | 0.18 | 0.179 |
| PLR | 32 | 0.19 | 0.295 | 0.11 | 0.404 |
| SII | 32 | 0.29 | 0.112 | 0.21 | 0.130 |

IL-1β, interleukin-1β; IL-4, interleukin-4; IL-6, interleukin-6; IFN-γ, interferon-γ; TNF-α, tumor necrosis factor-α; NK Cells, Natural Killer Cells; PNI, prognostic nutritional index; NLR, neutrophil-to-lymphocyte ratio; PLR, platelet-to-lymphocyte ratio; SII, systemic immune-inflammation index.

**Table S3. Peripheral immune and** **hematologic biomarkers in the PN group and non-PN group at** **baseline.**

| Haematological indicators | PN | | non-PN | | p |
| --- | --- | --- | --- | --- | --- |
|  | n | mean ± sd | n | mean ± sd |  |
| IL-1β | - | - | 1 | - | - |
| IL-4 | 14 | 1.86±1.66 | 4 | 3.93±3.51 | 0.328 |
| IL-6 | 14 | 15.79±18.21 | 4 | 9.92±4.58 | 0.959 |
| IFN-γ | 14 | 3.35±2.10 | 4 | 19.54±22.43 | 0.245 |
| TNF-α | 14 | 13.99±11.07 | 4 | 9.09±8.21 | 0.366 |
| Average Lymphocyte Count (/μL) | 15 | 1680.53±559.81 | 3 | 2118.00±335.72 | 0.216 |
| B Cells (%) | 15 | 9.87±3.41 | 3 | 9.53±4.78 | 0.884 |
| T Cells (%) | 15 | 71.19±7.64 | 3 | 66.17±2.63 | 0.287 |
| Treg/CD8⁺ T cell (%) | 15 | 26.06±5.62 | 3 | 21.23±11.02 | 0.426 |
| CD4⁺ T cells (%) | 15 | 40.20±8.31 | 3 | 40.40±9.91 | 0.970 |
| CD4⁺/Treg cell ratio | 15 | 1.68±0.76 | 3 | 2.33±1.28 | 0.498 |
| NK Cells (%) | 15 | 16.34±9.02 | 3 | 22.09±6.91 | 0.315 |
| Serum Albumin Level (g/L) | 18 | 41.53±4.17 | 9 | 42.33±4.11 | 0.639 |
| Neutrophil Count (×10⁹/L) | 18 | 4.45±1.71 | 10 | 3.61±1.97 | 0.375 |
| Lymphocyte Count (×10⁹/L) | 18 | 1.79±0.56 | 10 | 1.48±0.57 | 0.174 |
| sssPlatelet Count (×10⁹/L) | 18 | 289.50±69.06 | 10 | 281.80±62.13 | 0.772 |
| PNI | 18 | 50.48±4.99 | 9 | 50.03±5.43 | 0.834 |
| NLR | 18 | 2.77±1.46 | 10 | 2.65±1.55 | 0.796 |
| PLR | 18 | 177.46±70.32 | 10 | 219.72±111.73 | 0.229 |
| SII | 18 | 838.29±562.43 | 10 | 802.36±585.45 | 0.981 |

PN, received parenteral nutrition; non-PN, not received parenteral nutrition; IL-1β, interleukin-1β; IL-4, interleukin-4; IL-6, interleukin-6; IFN-γ, interferon-γ; TNF-α, tumor necrosis factor-α; NK Cells, Natural Killer Cells; PNI, prognostic nutritional index; NLR, neutrophil-to-lymphocyte ratio; PLR, platelet-to-lymphocyte ratio; SII, systemic immune-inflammation index.

**Table S4. Peripheral immune and hematologic biomarkers in the PN group and non-PN group at** **pre****operative.**

| Haematological indicators | PN | | non-PN | | p |
| --- | --- | --- | --- | --- | --- |
|  | n | mean ± sd | n | mean ± sd |  |
| IL-1β | 7 | 5.27±4.58 | 3 | 1.16±1.72 | 0.180 |
| IL-4 | 18 | 1.70±1.89 | 8 | 1.81±1.70 | 0.802 |
| IL-6 | 18 | 9.87±13.27 | 8 | 9.97±5.94 | 0.232 |
| IFN-γ | 18 | 16.70±38.71 | 8 | 5.43±4.97 | 0.559 |
| TNF-α | 18 | 8.27±9.23 | 8 | 8.47±5.26 | 0.577 |
| Average Lymphocyte Count (/μL) | 20 | 1564.60±510.10 | 7 | 1367.43±421.21 | 0.369 |
| B Cells (%) | 20 | 6.48±3.02 | 7 | 6.10±3.13 | 0.776 |
| T Cells (%) | 20 | 73.65±8.79 | 7 | 69.76±12.94 | 0.382 |
| Treg/CD8⁺ T cell (%) | 20 | 29.29±7.68 | 7 | 23.92±9.93 | 0.152 |
| CD4⁺ T cells (%) | 20 | 39.34±10.52 | 7 | 41.26±8.67 | 0.669 |
| CD4⁺/Treg cell ratio | 20 | 1.49±0.73 | 7 | 2.04±0.95 | 0.268 |
| NK Cells (%) | 20 | 18.18±9.65 | 7 | 22.21±12.49 | 0.386 |
| Serum Albumin Level (g/L) | 20 | 41.31±3.86 | 12 | 42.11±4.32 | 0.592 |
| Neutrophil Count (×10⁹/L) | 20 | 3.36±3.04 | 12 | 2.59±1.23 | 0.938 |
| Lymphocyte Count (×10⁹/L) | 20 | 1.68±0.49 | 12 | 1.29±0.38 | 0.024 |
| Platelet Count (×10⁹/L) | 20 | 152.45±42.56 | 12 | 168.58±56.52 | 0.366 |
| PNI | 20 | 49.73±3.57 | 12 | 48.57±4.95 | 0.450 |
| NLR | 20 | 2.15±2.08 | 12 | 2.16±1.30 | 0.552 |
| PLR | 20 | 95.88±30.01 | 12 | 137.57±48.83 | 0.005 |
| SII | 20 | 340.34±378.12 | 12 | 359.24±219.33 | 0.366 |

PN, received parenteral nutrition; non-PN, not received parenteral nutrition; IL-1β, interleukin-1β; IL-4, interleukin-4; IL-6, interleukin-6; IFN-γ, interferon-γ; TNF-α, tumor necrosis factor-α; NK Cells, Natural Killer Cells; PNI, prognostic nutritional index; NLR, neutrophil-to-lymphocyte ratio; PLR, platelet-to-lymphocyte ratio; SII, systemic immune-inflammation index.

**Table S5. Peripheral immune and hematologic biomarkers in the PN group and non-PN group at postoperative**

| Haematological indicators | PN | | non-PN | | p |
| --- | --- | --- | --- | --- | --- |
|  | n | mean ± sd | n | mean ± sd |  |
| IL-1β | 1 | - | 5 | 1.70±1.78 | - |
| IL-4 | 16 | 1.62±2.55 | 10 | 4.12±7.92 | 0.443 |
| IL-6 | 16 | 202.80±150.42 | 10 | 112.69±66.78 | 0.121 |
| IFN-γ | 16 | 4.31±6.07 | 10 | 4.97±4.73 | 0.476 |
| TNF-α | 16 | 9.23±6.25 | 10 | 5.75±5.03 | 0.102 |
| Average Lymphocyte Count (/μL) | 0 | - | 2 | 1399.50±924.19 | - |
| B Cells (%) | 1 | - | 2 | 3.95±1.68 | - |
| T Cells (%) | 1 | - | 2 | 60.76±4.34 | - |
| Treg/CD8⁺ T cell (%) | 1 | - | 2 | 22.36±2.68 | - |
| CD4⁺ T cells (%) | 1 | - | 2 | 33.98±5.89 | - |
| CD4⁺/Treg cell ratio | 1 | - | 2 | 1.52±0.08 | - |
| NK Cells (%) | 1 | - | 2 | 31.89±1.03 | - |
| Serum Albumin Level (g/L) | 21 | 34.03±3.37 | 12 | 34.30±4.49 | 0.845 |
| Neutrophil Count (×10⁹/L) | 21 | 8.35±2.78 | 12 | 8.15±3.68 | 0.671 |
| Lymphocyte Count (×10⁹/L) | 21 | 1.25±0.54 | 12 | 1.04±0.44 | 0.264 |
| Platelet Count (×10⁹/L) | 21 | 176.24±51.19 | 12 | 143.17±37.97 | 0.085 |
| PNI | 21 | 40.28±4.04 | 12 | 39.51±4.91 | 0.630 |
| NLR | 21 | 8.79±6.42 | 12 | 8.89±6.01 | 0.868 |
| PLR | 21 | 168.83±82.07 | 12 | 159.49±76.94 | 0.750 |
| SII | 21 | 1507.93±971.34 | 12 | 1347.71±1,132.15 | 0.405 |

PN, received parenteral nutrition; non-PN, not received parenteral nutrition; IL-1β, interleukin-1β; IL-4, interleukin-4; IL-6, interleukin-6; IFN-γ, interferon-γ; TNF-α, tumor necrosis factor-α; NK Cells, Natural Killer Cells; PNI, prognostic nutritional index; NLR, neutrophil-to-lymphocyte ratio; PLR, platelet-to-lymphocyte ratio; SII, systemic immune-inflammation index.

**Table S6. Circulating inflammatory and immune-related parameters in patients achieving MPR and non-MPR at baseline.**

| Haematological indicators | MPR | | non-MPR | | p |
| --- | --- | --- | --- | --- | --- |
|  | n | mean ± sd | n | mean ± sd |  |
| IL-1β | 0 | - | 1 | - | - |
| IL-4 | 6 | 1.80±1.64 | 12 | 2.59±2.53 | 0.605 |
| IL-6 | 6 | 14.37±17.25 | 12 | 14.54±16.49 | 0.820 |
| IFN-γ | 6 | 3.02±2.38 | 12 | 8.91±14.19 | 0.373 |
| TNF-α | 6 | 8.22±4.43 | 12 | 15.24±11.97 | 0.349 |
| Average Lymphocyte Count (/μL) | 7 | 1848.00±594.31 | 11 | 1693.27±535.69 | 0.575 |
| B Cells (%) | 7 | 10.81±4.08 | 11 | 9.18±3.13 | 0.353 |
| T Cells (%) | 7 | 68.07±7.25 | 11 | 71.81±7.21 | 0.300 |
| Treg/CD8⁺ T cell (%) | 7 | 27.67±3.09 | 11 | 23.71±7.87 | 0.156 |
| CD4⁺ T cells (%) | 7 | 36.79±7.83 | 11 | 42.43±8.13 | 0.328 |
| CD4⁺/Treg cell ratio | 7 | 1.36±0.36 | 11 | 2.06±0.97 | 0.126 |
| NK Cells (%) | 7 | 18.82±10.47 | 11 | 16.33±7.95 | 0.574 |
| Serum Albumin Level (g/L) | 11 | 39.70±4.42 | 16 | 43.24±3.25 | 0.024 |
| Neutrophil Count (×10⁹/L) | 11 | 4.10±1.85 | 17 | 4.18±1.85 | 0.906 |
| Lymphocyte Count (×10⁹/L) | 11 | 1.83±0.57 | 17 | 1.58±0.57 | 0.204 |
| Platelet Count (×10⁹/L) | 11 | 270.00±65.83 | 17 | 297.59±65.09 | 0.285 |
| PNI | 11 | 48.86±5.67 | 16 | 51.34±4.47 | 0.217 |
| NLR | 11 | 2.38±1.35 | 17 | 2.95±1.53 | 0.244 |
| PLR | 11 | 160.31±60.76 | 17 | 213.41±97.51 | 0.147 |
| SII | 11 | 692.46±504.66 | 17 | 911.52±591.79 | 0.244 |

IL-1β, Interleukin-1β; IL-4, Interleukin-4; IL-6, Interleukin-6; IFN-γ, Interferon-γ; TNF-α, Tumor Necrosis Factor-α; NK Cells, Natural Killer Cells; PNI, Prognostic Nutritional Index; NLR, Neutrophil-to-Lymphocyte Ratio; PLR, Platelet-to-Lymphocyte Ratio; SII, Systemic Immune-Inflammation Index.

**Table S7.** **Circulating inflammatory and immune-related parameters in patients achieving MPR and non-MPR at preoperative.**

| Haematological indicators | MPR | | non-MPR | | p |
| --- | --- | --- | --- | --- | --- |
|  | n | mean ± sd | n | mean ± sd |  |
| IL-1β | 3 | 9.18±3.42 | 7 | 1.84±2.26 | 0.021 |
| IL-4 | 8 | 1.78±1.66 | 18 | 1.71±1.91 | 0.436 |
| IL-6 | 8 | 7.08±4.86 | 18 | 11.15±13.25 | 0.657 |
| IFN-γ | 8 | 9.17±16.14 | 18 | 15.04±37.84 | 0.436 |
| TNF-α | 8 | 5.68±5.30 | 18 | 9.52±8.96 | 0.242 |
| Average Lymphocyte Count (/μL) | 9 | 1669.56±587.68 | 18 | 1435.44±428.28 | 0.248 |
| B Cells (%) | 9 | 5.68±3.73 | 18 | 6.73±2.60 | 0.194 |
| T Cells (%) | 9 | 70.68±9.98 | 18 | 73.62±10.01 | 0.631 |
| Treg/CD8⁺ T cell (%) | 9 | 32.64±8.43 | 18 | 25.52±7.63 | 0.037 |
| CD4⁺ T cells (%) | 9 | 33.76±9.15 | 18 | 42.87±9.09 | 0.022 |
| CD4⁺/Treg cell ratio | 9 | 1.11±0.42 | 18 | 1.89±0.85 | 0.016 |
| NK Cells (%) | 9 | 21.64±11.81 | 18 | 18.02±9.69 | 0.402 |
| Serum Albumin Level (g/L) | 11 | 40.52±5.04 | 21 | 42.18±3.30 | 0.269 |
| Neutrophil Count (×10⁹/L) | 11 | 3.21±2.83 | 21 | 3.00±2.41 | 0.890 |
| Lymphocyte Count (×10⁹/L) | 11 | 1.72±0.46 | 21 | 1.44±0.48 | 0.127 |
| Platelet Count (×10⁹/L) | 11 | 154.27±59.90 | 21 | 160.71±42.03 | 0.725 |
| PNI | 11 | 49.10±5.56 | 21 | 49.39±3.25 | 0.968 |
| NLR | 11 | 2.14±2.39 | 21 | 2.16±1.47 | 0.481 |
| PLR | 11 | 93.74±41.95 | 21 | 120.83±40.92 | 0.042 |
| SII | 11 | 341.11±414.48 | 21 | 350.74±276.56 | 0.238 |

IL-1β, interleukin-1β; IL-4, interleukin-4; IL-6, interleukin-6; IFN-γ, interferon-γ; TNF-α, tumor necrosis factor-α; NK Cells, Natural Killer Cells; PNI, prognostic nutritional index; NLR, neutrophil-to-lymphocyte ratio; PLR, platelet-to-lymphocyte ratio; SII, systemic immune-inflammation index.

**Table S8.** **Circulating inflammatory and immune-related parameters in patients achieving MPR and non-MPR at postoperative.**

| Haematological indicators | MPR | | non-MPR | | p |
| --- | --- | --- | --- | --- | --- |
|  | n | mean ± sd | n | mean ± sd |  |
| IL-1β | 1 | - | 5 | 1.70±1.78 | - |
| IL-4 | 7 | 2.58±3.58 | 19 | 2.58±5.88 | 0.706 |
| IL-6 | 7 | 119.57±79.36 | 19 | 186.04±143.20 | 0.209 |
| IFN-γ | 7 | 4.71±7.41 | 19 | 4.50±4.87 | 0.643 |
| TNF-α | 7 | 8.30±4.68 | 19 | 7.74±6.48 | 0.369 |
| Average Lymphocyte Count (/μL) | 0 | - | 2 | 1,399.50±924.19 | - |
| B Cells (%) | 1 | - | 2 | 3.95±1.68 | - |
| T Cells (%) | 1 | - | 2 | 60.76±4.34 | - |
| Treg/CD8⁺ T cell (%) | 1 | - | 2 | 22.36±2.68 | - |
| CD4⁺ T cells (%) | 1 | - | 2 | 33.98±5.89 | - |
| CD4⁺/Treg cell ratio | 1 | - | 2 | 1.52±0.08 | - |
| NK Cells (%) | 1 | - | 2 | 31.89±1.03 | - |
| Serum Albumin Level (g/L) | 12 | 33.89±4.12 | 21 | 34.26±3.62 | 0.790 |
| Neutrophil Count (×10⁹/L) | 12 | 7.69±2.36 | 21 | 8.61±3.44 | 0.418 |
| Lymphocyte Count (×10⁹/L) | 12 | 1.36±0.40 | 21 | 1.07±0.54 | 0.054 |
| Platelet Count (×10⁹/L) | 12 | 153.83±33.46 | 21 | 170.14±55.80 | 0.432 |
| PNI | 12 | 40.68±3.86 | 21 | 39.61±4.60 | 0.286 |
| NLR | 12 | 6.35±2.93 | 21 | 10.24±7.12 | 0.048 |
| PLR | 12 | 124.29±51.90 | 21 | 188.94±83.35 | 0.018 |
| SII | 12 | 955.71±478.03 | 21 | 1,731.93±1,141.28 | 0.033 |

IL-1β, interleukin-1β; IL-4, interleukin-4; IL-6, interleukin-6; IFN-γ, interferon-γ; TNF-α, tumor necrosis factor-α; NK Cells, Natural Killer Cells; PNI, prognostic nutritional index; NLR, neutrophil-to-lymphocyte ratio; PLR, platelet-to-lymphocyte ratio; SII, systemic immune-inflammation index.
